# The radiological outcome in lumbar interbody fusion among rheumatoid arthritis patients: a 20-year retrospective study

**DOI:** 10.1186/s12891-021-04531-y

**Published:** 2021-08-05

**Authors:** Kuan-Kai Tung, Yun-Che Wu, Kun-Hui Chen, Chien-Chou Pan, Wen-Xian Lu, Ning-Chien Chin, Cheng-Min Shih, Fang-Wei Hsu, Cheng-Hung Lee

**Affiliations:** 1 Department of Orthopedics, Taichung Veterans General Hospital, Taichung, Taiwan; 2 Department of Biomedical Engineering, Hung Kuang University, Taichung, Taiwan; 3 Department of Computer Science and Information Engineering, Providence University, Taichung, Taiwan; 4 Department of Nursing, Jenteh Junior College of Medicine, Nursing and Management, Miaoli, Taiwan; 5 Department of Rehabilitation Science, Jenteh Junior College of Medicine, Nursing and Management, Miaoli, Taiwan; 6 Department of Orthopedics, Feng Yuan Hospital Ministry of Health and Welfare, Taichung, Taiwan; 7 Department of Physical Therapy, Hung Kuang University, Taichung, Taiwan; 8 PhD Degree Program of Biomedical Science and Engineering, College of Biological Science and Technology, National Yang Ming Chiao Tung University, Hsinchu, Taiwan; 9 Department of Orthopedics, Kuang Tien General Hospital, Taichung, Taiwan; 10 National Chung Hsing University, Taichung, Taiwan; 11 Department of Food Science and Technology, Hung Kuang University, Taichung, Taiwan

**Keywords:** RA, ALIF, OLIF, TLIF, Short lumbar spinal fusion, Radiographic outcome

## Abstract

**Background:**

Clinical outcomes amongst Rheumatoid Arthritis (RA) patients have shown satisfactory results being reported after lumbar surgery. The increased adoption of the interbody fusion technique has been due to a high fusion rate and less invasive procedures. However, the radiographic outcome for RA patients after receiving interbody fusion has scarcely been addressed in the available literature.

**Methods:**

Patients receiving interbody fusion including ALIF, OLIF, and TLIF were examined for implant cage motion and fusion status at two-year follow-up. Parameters for the index correction level including ADH, PDH, WI, SL, FW, and FH were measured and compared at pre-OP, post-OP, and two-year follow-up.

**Results:**

We enrolled 64 RA patients at 104 levels (mean 64.0 years old, 85.9% female) received lumbar interbody fusion. There were substantial improvement in ADH, PDH, WI, SL, FW, and FH after surgery, with both ADH and PDH having significantly dropped at two-year follow up. The OLIF group suffered from a higher subsidence rate with no significant difference in fusion rate when compared to TLIF. The fusion rate and subsidence rate for all RA patients was 90.4 and 28.8%, respectively.

**Conclusions:**

We revealed the radiographic outcomes of lumbar interbody fusions towards symptomatic lumbar disease in RA patients with good fusion outcome despite the relative high subsidence rate amongst the OLIF group. Those responsible for intra-operative endplate management should be more cautious to avoid post-OP cage subsidence.

## Introduction

Rheumatoid Arthritis (RA) is one of the most prevalent chronic inflammatory diseases causing structural changes, including major joint deformity and soft tissue damage. The axial skeleton come as the third most involved location of RA after the hands and feet [1]. Involvement of the cervical spine is much common than that of the lumbar spine, affecting 44 to 88% of RA patients [1, 2]. However, a 45% comparatively high frequency of lumbar lesions has been reported amongst patients who have suffered from more than ten years of RA [3]. Atlantoaxial dislocation and subaxial subluxation may both serve as end-stage complications of RA in the cervical spine [4]. On the other hand, lumbar lesions in patients with RA have been presented by subluxation and disc narrowing, with vertebral osteophytosis, apophyseal destruction, and osteoporosis being less common [5–7]. The facet joints of the lumbar spine are considered the most effected location, with synovitis and degenerative changes resulting in pain and disability [3, 8]. Although the discovertebral junction is not a synovial joint, enthesopathy destroys the collagen fiber of the endplate, which eventually leads to erosion, loss of disk space, and instability [9, 10]. Overall incidence of endplate erosion is 70.6% in RA [9]. Additionally, the positive association between cervical and lumbar spinal lesions has been addressed. By evaluating the sagittal T1-weighted Magnetic Resonance Imaging (MRI) among 201 patients, 56.2% patients were presented with end plate erosion amongst the RA patients [11].

The ideal treatment for lumbar lesions in RA has been recently discussed. Satisfactory self-reported and radiographic outcomes have been reported [12–15] by those having undergone lumbar fusion. Recently, the introduction and increased adoption of the Lumbar Interbody Fusions (LIFs) technique has proven its efficacy for restoring lumbar lordosis, index disc height and the central canal and foraminal areas through the insertion of lordotic angular grafts in a minimally invasive assessment [15]. However, radiographic fusion efficacy has not been reported among RA patients. Therefore, in this study, we aim to retrospectively evaluate radiographic outcomes after RA patients have received interbody spinal fusion for symptomatic lumbar disease.

## Materials and methods

### Study population

All RA patients were enrolled from Taichung Veterans General Hospital who had received lumbar interbody fusion for symptomatic lumbar disorder from the years 2000 to 2021. All patients underwent both interbody fusion and posterior instrumentation. The selection criteria for the patients were: (1) the presence of low back pain or sciatia, and unresponsive to conservative treatment for more than 6 months; (2) the patient’s pre-operative (pre-OP) and post-operative (post-OP) clinical imaging data and follow-up records being complete; and (3) patient was diagnosed with RA before the surgery for more than 10 years. Exclusion criteria included: (1) loss during follow-up; (2) spinal deformity due to the presence of an active infection, malignancy, trauma, or neuromuscular disease etiology (3) patient receiving previous lumbar surgery; (4) patient without full-length lateral spine radiographs at pre-OP, post-OP, and two-year follow-up. Diagnosis of disease was based upon the International Classification of Diseases, Ninth Revision, Clinical Modification (ICD-9-CM). The RA cohort was defined as: (1) patients diagnosed with RA for more than ten years through the catastrophic illness card before the operation; (2) patients were followed from their index date to date of surgery, death, withdrawal from the hospital database, or the end of the year 2021, whichever came first. The diagnosis of RA, defined by the catastrophic illness card during the study period, was certified by rheumatologists according to the criteria of American College of Rheumatology in 1987 (ACR 1987) [16].

### Surgical methods

Three interbody fusion techniques were included in this study: (1) Transforaminal Lateral Interbody Fusion (TLIF); (2) Anterior Lateral Interbody Fusion (ALIF); and (3) Oblique Lateral Interbody Fusion (OLIF). TLIF was performed by paramedian mini-open incision in prone position. The index disc levels of L1-S1 were exposed and unilateral laminectomy with inferior facetectomy was performed. Bone grafts and implant cages were inserted under sufficient exposure; ALIF was performed by paramedian (all level) or Mini-Pfannestiel (L5/S1) incision in supine position whilst patients receiving OLIF was prepared in right-lateral position. Blunt dissection with secure of vessels and abdominal organs for exposure of retroperitoneal corridor was performed. Annulus fibrosus of index correction levels were released. Implant cages were inserted under sufficient exposure. In all three procedures, position of the implants were confirmed through x-ray fluoroscopy. Adequate hemostasis was performed after closure of the wound.

### Radiographic assessment

**Fig. 1:**
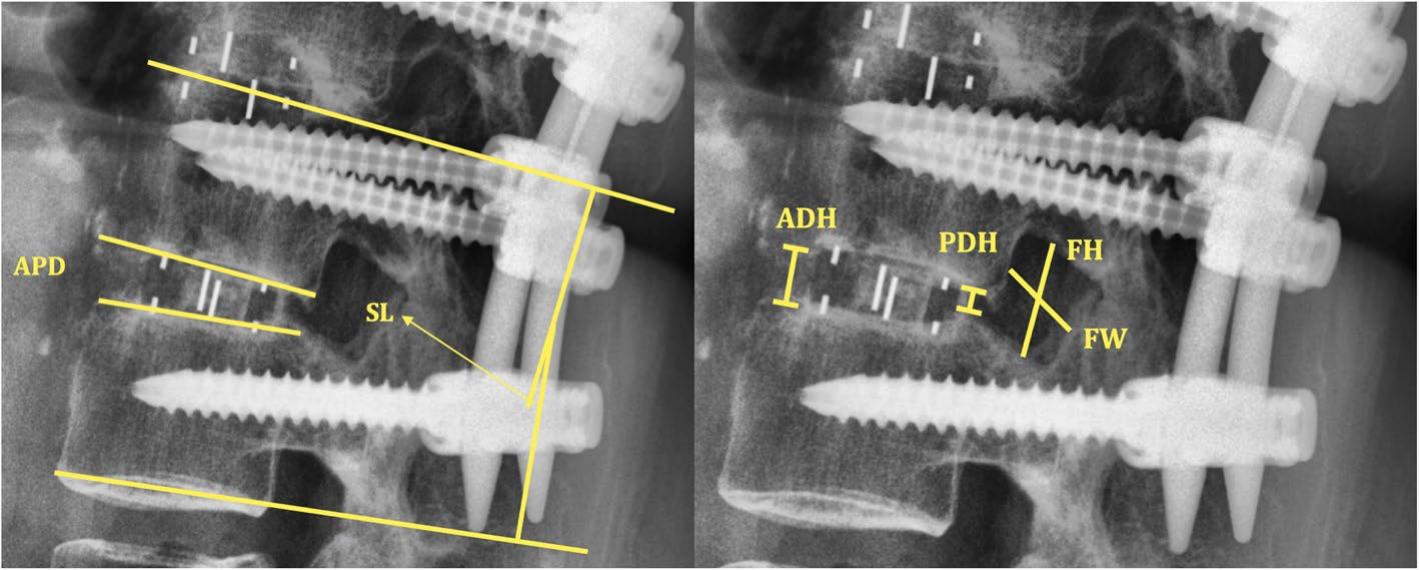
Schematic of radiographic measurement. APD, Anterior–Posterior Diameter; SL, Segmental Lordosis; ADH, Anterior Disc Height; PDH, Posterior Disc Height; FH, Foraminal Height; FW, Foraminal Width

In order to evaluate fusion status via motion, lateral spine flexion–extension radiographs at the patient’s pre-OP visit, nearest post-OP follow-up, and latest follow-up were all measured and analyzed by K-KT and W-CW using validated Surgimap surgical planning software (Nemaris Inc., New York, NY, United States) [17]. All radiographic measurements were performed while being positioned at a central location based upon standardized techniques, including: (1) Anterior–posterior Diameter (APD): defined as the mean length of the superior inferior vertebral endplate and inferior superior endplate [18]; (2) Anterior Disc Height (ADH): “measured in the planes of the anterior surfaces of the adjacent vertebral bodies, where the distances between the adjacent superior and inferior end plates were the shortest.” [19]; (3) Posterior Disc Height (PDH): “measured in the planes of the posterior surfaces of the adjacent vertebral bodies, where the distances between the adjacent superior and inferior end plates were the shortest.” [19]; (4) Foraminal Width (FW): “measured as the shortest distance between the superior edge of the superior articular process of the caudal vertebra and the posterior edge of inferior endplate of the cranial vertebra.” [20]; (5) Foraminal Height (FH): “measured as the maximum distance between the inferior margin of the pedicle of the superior vertebra and the superior margin of the pedicle of the inferior vertebra.” [20]; (6) Segmental Lordosis (SL): “the angle subtended by the superior endplate line and the inferior endplate line of a segment with an interbody cage. However, the SL at L5-S1 was measured as the angle subtended by the superior endplate line of L5 and the superior endplate line of S1” [21], and (7) Wedge Index (WI): Calculated by ADH-PDH/APD [18] (Fig. 1).

### Fusion outcomes

**Fig. 2:**
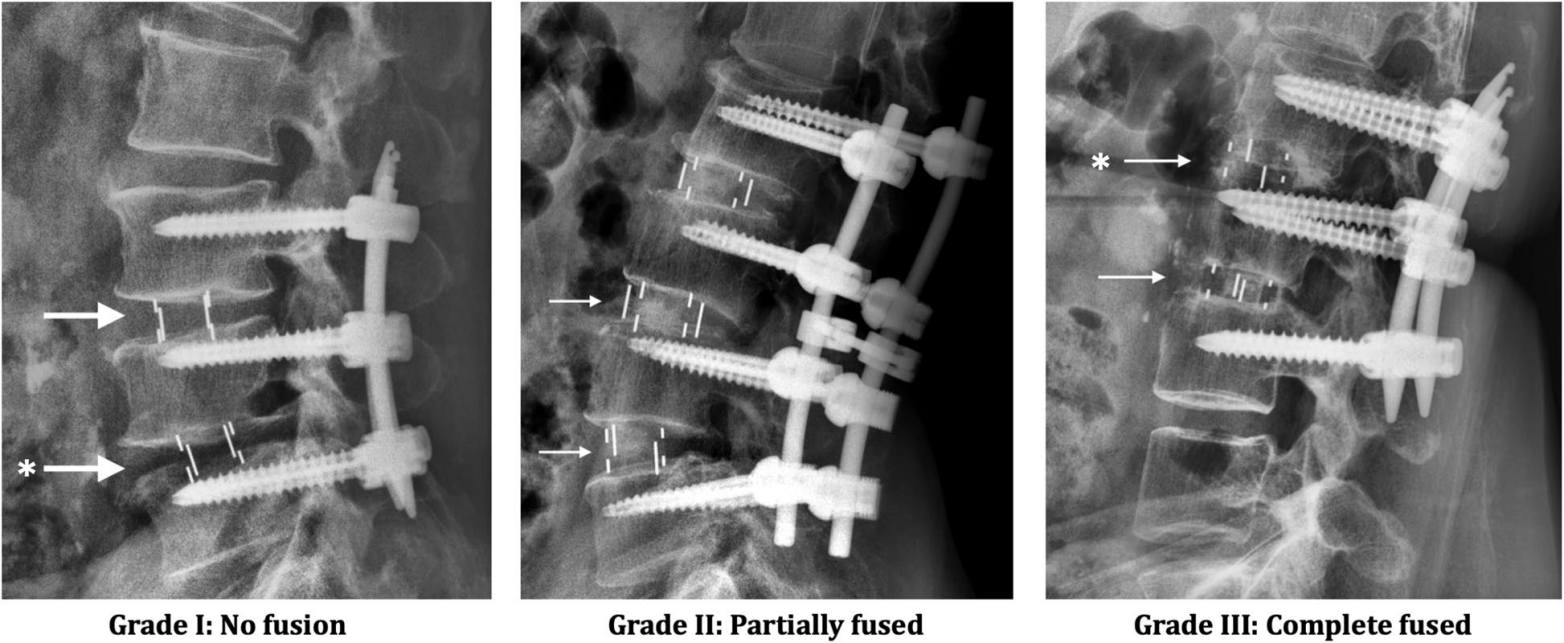
Grading for fusion status. Arrow ( →), interbody cage in position. Asterisk (*), the subsidence of LIF cage. Noted that cage subsidence can be observed in different fusion status

K-KT and W-CW reviewed each of the flexion–extension plain films (unblinded) and recorded fusion status. Each fusion level was evaluated separately by Hutter method [22] according to the Santos criteria [23, 24] of fusion grading at 2 year follow-up: (1) Grade I: No fusion. Any motion or radiolucency around the device; (2) Grade II: Partially fused. No motion around the device without definite bony opacity formation in/around the cage; (3) Grade III: Complete fused: No motion or radiolucency around the device with definite bony opacity formation in/around the cage (Fig. 2). Fusion rate was calculated by the proportion of grade II and grade III amongst study cohort. Interbody cage subsidence was defined as sinking of interbody cage due to progression endplate collapse ≧2 mm after the operation.

### Statistical analyses

Demographic parameters were included in our analysis. Normality of data was determined via the Shapiro–Wilk test. Continuous variables were described through mean and standard deviation. The comparison between LIFs was conducted by one-way analysis of variance (ANOVA), Independent Sample t test, Wilcoxon rank-sum test, or Chi-square test according to appropriate models. Change in parameter was conducted via generalized estimating equation (GEE). All statistical analyses were performed using SPSS version 24 (IBM, Armonk, New York, USA). Two-sided *p*-values < 0.05 were considered statistically significant.

## Results

### Patient population demographics

We enrolled 64 patients at 104 levels with RA receiving lumbar interbody fusion for lumbar disease from 2000 to 2021. The demographic data of the patients are presented in Table 1. Amongst the patients, 85.9% were female with mean age of 64 ± 5.3 years. Most of the patient were operated under the diagnosis of spondylolisthesis. Nine patient suffered from spondylosis whilst three patient were diagnosed as non-traumatic compression fracture. Major operative level of LIFs were located in level L3 to S1, with no more than four levels of spinal fusion. The fusion rate and subsidence rate of all RA patients was 90.4 and 28.8%. Two patients received revision operation due to complication of Adjacent Segment Disease (ASD) [25] in the OLIF group and unstable fixation of the pedicle screw in the TLIF group, respectively.

**Table 1:** Demographic data of the study population stratified by lumbar interbody fusion types

	**ALIF**	**OLIF**	**TLIF**	**Overall**	***P* value**
Patient number	3	20	41	64	
Correction levels	3	44	57	104	
Age	67 ± 1.7	66.5 ± 4.2	64.7 ± 5.5	64 ± 5.3	0.57
Female gender (%)	100%	85%	85.4%	85.9%	0.6
Pre−OP diagnosis					
Spondylolisthesis	1	15	34	50	
Spondylosis	2	5	6	13	
Other	0	0	1	1	
Index fusion level					0.09
L1-L2	0	1	0	1	
L2-L3	0	5	3	8	
L3-L4	1	18	13	32	
L4-L5	1	20	30	51	
L5-S1	1	0	11	12	
Fusion status					0.8
Grade I	0	5	5	10	
Grade II	0	10	7	17	
Grade III	3	29	45	77	
Fusion rate (%) (Grade II and III)	100	88.6	91.2	90.4	
Subsidence (%)	0	40.9	21.1	28.8	**0.03**

*P* value < 0.05 was consider statistically significant between OLIF and TLIF. Values expressed as the mean ± standard deviation. Boldface type indicates statistical significance

### Patients radiographic result

**Fig. 3:**
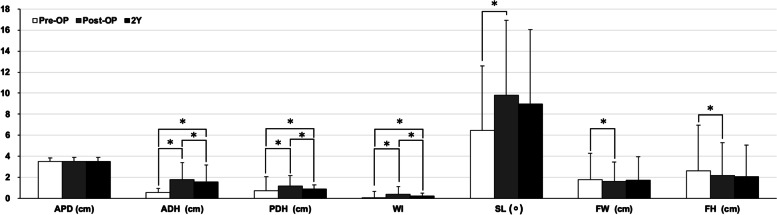
Radiographic outcome measured at pre-OP, post-OP, and two-year follow-up amongst all RA patient. *, statistically significant between measurements (*p* < 0.05). APD, Anterior–Posterior Diameter; ADH, Anterior Disc Height; PDH, Posterior Disc Height; WI, Wedge Index; FH, Foraminal Height; FW, Foraminal Width

The radiographic result of all patients were presented in Fig. 3. The ADH and PDH showed significant increase after operation (from 0.6 ± 0.4 to 1.8 ± 1.6 cm in ADH, *p* < 0.05; from 0.7 ± 1.3 to 1.2 ± 1 in cm PDH, *p* < 0.05). However, both parameters dropped at two-year follow-up (1.6 ± 1.6 cm in ADH and 0.9 ± 0.4 cm in PDH, *p* < 0.05). The WI showed similar trend comparing to ADH and PDH (pre-OP: 0.2 ± 0.8, post-OP: 0.4 ± 1.0, and 0.4 ± 0.6 at two-year follow-up). The SL (from 6.4 ± 6.1 to 9.8 ± 7.2 degree, *p* < 0.05), FW (from 1.8 ± 2.5 to 1.6 ± 1.8 cm, *p* < 0.05), and FH (from 2.6 ± 4.3 to 2.2 ± 3.1 cm, *p* < 0.05) demonstrated significant perioperative changes. However, these changes were diminished at two-year follow-up. There were no statistically significant change in the APD during the follow-up.

### Comparison of ALIF, OLIF, and TLIF

**Fig. 4:**
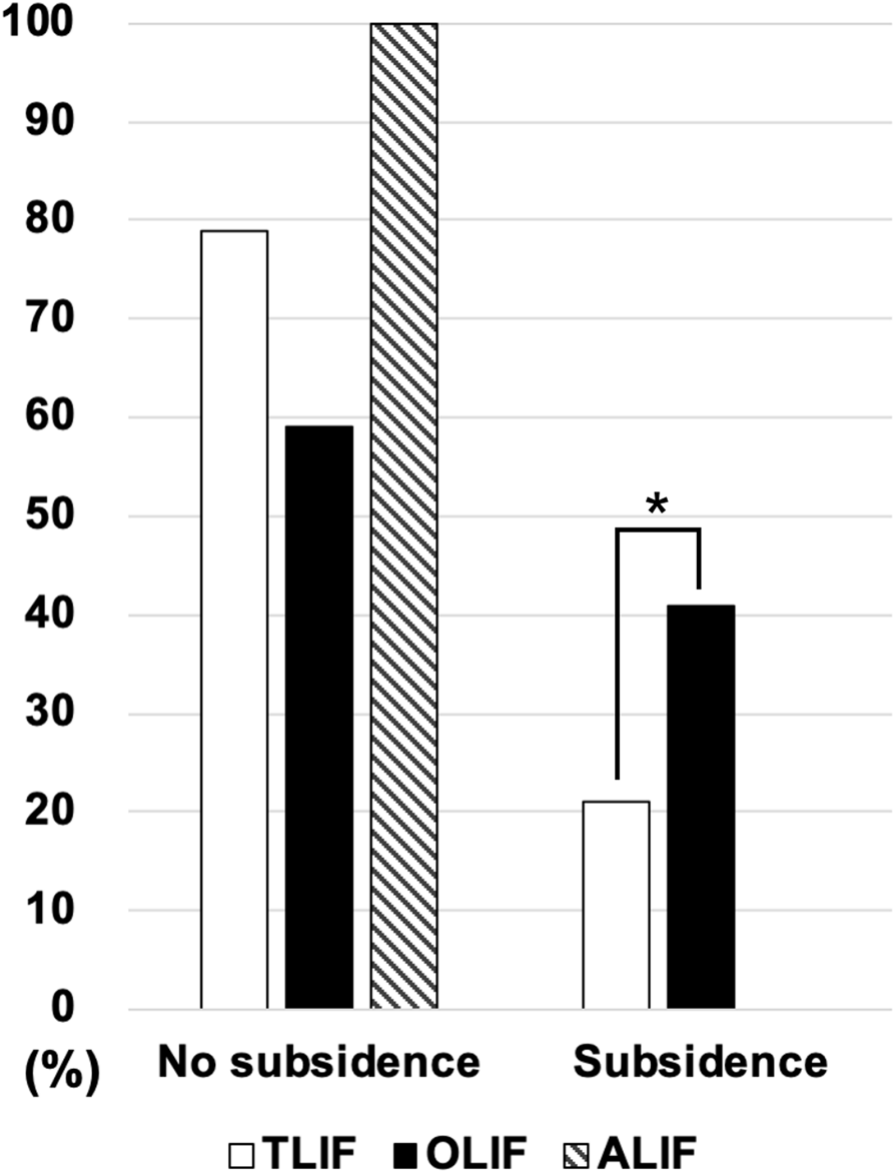
Comparison of cage subsidence rate amongst ALIF, OLIF, and TLIF. *, the OLIF group had statistically significant higher subsidence rate comparing to TLIF group (*p* < 0.05). ALIF, Anterior Interbody Lumbar Fusion; OLIF, Oblique Interbody Lumbar Fusion; TLIF, Transforaminal Interbody Lumbar Fusion

Since the unequal disturbance of patient numbers in three groups, the statistical comparison was made between only in the OLIF and TLIF group. We only explain the ALIF group in description without making a statistical inference. There were three, 20, 41 patients at three, 44, and 57 levels receiving ALIF, OLIF, and TLIF, respectively. The comparison of demographic data and radiographic outcomes of three group were demonstrated in Tables 1 and 2. The cage subsidence rate was 0, 21, and 40.9% amongst the patients receiving ALIF, OLIF, and TLIF (Fig. 4). The OLIF group had statistically significant higher subsidence rate comparing to TLIF group (40.9 to 21.1%, *p* < 0.05). The fusion rate was 100, 88.6, and 91.2% amongst the patients receiving ALIF, OLIF, and TLIF. All three patients in the ALIF group were complete fused (Grade III) without subsidence at two-year follow-up. Patients were all female gender and had received ALIF under the diagnosis of spondylolisthesis and spondylosis. There was no statistical difference in preoperative diagnosis, index fusion level, fusion status distribution, and fusion rate between OLIF and TLIF group (Table 1).

**Table 2:** Radiographical outcome of the study population stratified by lumbar interbody fusion types

	**ALIF**	**OLIF**	**TLIF**	**Overall**	***P* value**
APD					
Pre-OP	3.3 ± 0.4	3.4 ± 0.3	3.5 ± 0.4	3.5 ± 0.4	0.27
Post-OP	3.4 ± 0.5	3.3 ± 0.3	3.6 ± 0.5	3.5 ± 0.4	0.08
2Y	3.2 ± 0.2	3.4 ± 0.3	3.6 ± 0.4	3.5 ± 0.4	0.05
ADH					
Pre-OP	0.7 ± 0.5	0.4 ± 0.3	0.7 ± 0.4	0.6 ± 0.4	0.05
Post-OP	1.7 ± 0.3	2.3 ± 2.3	1.3 ± 0.3	1.8 ± 1.6	** < 0.01**
2Y	1.7 ± 0.1	2.1 ± 2.3	1.1 ± 0.3	1.6 ± 1.6	** < 0.01**
PDH					
Pre-OP	0.3 ± 0.1	1.1 ± 2	0.5 ± 0.3	0.7 ± 1.3	0.28
Post-OP	1.2 ± 0.6	1.6 ± 1.4	0.8 ± 0.3	1.2 ± 1	** < 0.01**
2Y	0.9 ± 0.9	1.1 ± 0.5	0.7 ± 0.3	0.9 ± 0.4	** < 0.01**
WI					
Pre-OP	0.2 ± 0.5	0.2 ± 0.7	0.3 ± 0.4	0.2 ± 0.8	**< 0.01**
Post-OP	0.4 ± 0.7	0.3 ± 1.8	0.4 ± 0.8	0.4 ± 1.0	**< 0.01**
2Y	0.4 ± 0.6	0.3 ± 0.2	0.4 ± 0.4	0.4 ± 0.6	** < 0.01**
SL					
Pre-OP	3.2 ± 0.5	5.6 ± 4.4	7.1 ± 7.2	6.4 ± 6.1	0.28
Post-OP	9.1 ± 12.6	8 ± 6.1	11.3 ± 7.6	9.8 ± 7.2	**0.02**
2Y	8.4 ± 8	7.5 ± 6.3	10.1 ± 7.5	8.9 ± 7.1	0.09
FW					
Pre-OP	1.2 ± 0.2	1.5 ± 0.6	1.1 ± 0.3	1.8 ± 2.5	0.16
Post-OP	1.3 ± 0.6	2.1 ± 2.7	1.3 ± 0.3	1.6 ± 1.8	**0.04**
2Y	1.5 ± 0.8	2.4 ± 3.3	1.3 ± 0.4`	1.7 ± 2.2	**0.03**
FH					
Pre-OP	1.8 ± 0.3	3.2 ± 6.3	1.4 ± 0.3	2.6 ± 4.3	0.05
Post-OP	2 ± 0.1	3 ± 4.6	1.6 ± 0.4	2.2 ± 3.1	**0.04**
2Y	1.5 ± 0.3	2.9 ± 4.4	1.5 ± 0.3	2.1 ± 3	**0.03**

*APD* anterior–posterior diameter, *ADH* anterior disc height, *PDH* posterior disc height, *WI* wedge index, *SL* segmental lordosis, *FW* foraminal width, *FH* foraminal height, *Y* year

*P* value < 0.05 was consider statistically significant between OLIF and TLIF. Values expressed as the mean ± standard deviation. Boldface type indicates statistical significance

With similar baseline disc heights, the OLIF group had greater ADH and PDH than the TLIF group at post-OP and two-year follow-up. Since there were no statistically difference in APD, the WI were affected by the difference between PDH and ADH. The SL were greater in the TLIF group after the operation. However, there were no statistically difference at two-year follow-up. The FH and FW were greater in the OLIF group at post-OP and two-year follow-up comparing to TLIF group (Table 2).

## Discussion

In this study, 64 patients at 104 levels with RA who had received lumbar interbody fusion for lumbar disease showed good radiological fusion rates at two-year follow-up. Among the patients, 85.9% were female and 90.4% of the surgical levels were at least partially fused (Grade II to III). The overall subsidence rate was 28.8%. There were substantial improvements in ADH, PDH, WI, SL, FW, and FH after surgery, with ADH and PDH having significantly dropped at two-year follow up. Despite patients receiving OLIF suffering from a significantly higher rate of cage subsidence compared to TLIF group, the fusion rate showed no significant difference between OLIF and TLIF. To our knowledge, this is the first study reporting radiographic fusion status among RA patients after having received LIFs.

The surgical outcomes and complications from lumbar lesions in RA patients have been shown to be highly associated with disease activity. In a study [26] involving patients receiving short lumbar fusion, patients with RA suffered from a 4.5 times higher risk of ASD. These patients required surgery more so than those without RA. Moreover, three-level correction caused a 2.7 times higher risk in patients developing ASD when compared to one- or two-level correction. In current study, only one patient suffered from ASD after receiving a four-level OLIF operation (L2 to S1). Posterolateral Lumbar Fusion (PLF) surgery demonstrated similar outcomes, with good-to-excellent results occurring in both RA and non-RA patients [12]. Another study [13] evaluating both the clinical and radiological outcomes of patients with RA and non-RA who were undergoing instrumented PLF revealed similar improvements after surgery, with 37.5% of the RA patients requiring revision surgery owing to both implant failure and post-operative infection. Alternatively, laminectomy or laminotomy without spinal fusion has been suggested for use by Seki et al. [14] due to their lower complication rates. RA patients who were receiving decompression and spinal fusion for lumbar spinal disorders suffered a greater revision rate and ASD occurrence. In their study, 58% of the patients underwent short lumbar fusion, which was similar to the demographic composition of the current study. The adoption of an interbody cage in our study demonstrated adequate sustainability and fusion outcomes. In the current study, 90.4% of the surgical levels were at least partially fused (Grade II and III, Table 1). Moreover, ADH, PDH, and WI showed significant improvement at two-year follow-up with only one patient received revision surgery in the OLIF group due to ASD. Upon biochemical examinations [27], PLF showed greater instability in posterior instruments as well as less stress peaks when compared to OLIF and TLIF. LIFs have demonstrated their efficacy towards the fusion rate in numerous reports found in the available literature [28–32]. Moreover, 94.2 and 97.9% successful fusion rates were seen in ALIF and OLIF, respectively, with an approximately 4.4% cage subsidence rate in OLIF amongst general population [29, 30]. However, in the current study, the cage subsidence rate at two-year follow up was 28.8%, which is much higher than general population that reported in the available literature.

In contrast to the cervical involvement of RA, lumbar pathology in the lumbar spine has been addressed infrequently in the available literature. Inflammatory arthropathy of the spine is often presented with both the destruction of facet joints and endplate erosion amongst RA patients [5–7]. A significantly higher incidence of apophyseal joint damage and stenosis of the vertebral body has been reported [5]. The severity of erosive and sclerotic changes, irregularity of the vertebral end plates, and collapse of the intervertebral discs or vertebral bodies have all served as prediction factors for surgical outcomes. Moreover, patients with more severe mutilating peripheral joint involvement appear to have a higher frequency of lumbar lesions [3]. In TLIF, cage migration and retropulsion are affected by osteoporosis, a pear-shaped intervertebral disc, posterior positioning of the cage, and the presence of endplate injury. These risk factors may lead to a poor fusion rate and cage subsidence [33]. However, it is difficult to obtain the medication history of these patients in the current study. Major challenges for RA-related osteopenia may result from the primary effect of the disease’s nature, menopause, or being secondary to the effect of anti-inflammatory agents. Complications including ASD, non-union, and instability have been common reasons for osteopenia-related instrumentation failure amongst RA patients [12]. In the current study, we found a significantly higher cage subsidence rate amongst OLIF patients when compared to the TLIF groups. According to the operative methods, insufficient release of the annulus fibrosus and a larger fusion cage may cause additional endplate damage, resulting in a higher rate of cage subsidence. However, a greater contact surface provided a better medium for bone fusion. In this study, we found no difference in fusion rates between OLIF, and TLIF patients. Thus, despite the higher cage subsidence rate (40.9%), the fusion rate of OLIF (88.6%) has proven its efficacy among RA patients. In ALIF group, all three patients were successfully fused during the follow-up. We suggest that medical responsible for intra-operative management of the endplate be more cautious to avoid post-OP cage subsidence and iatrogenic endplate injury whilst conducting OLIF. The concept for evaluating bone condition when seeking adequate patient selection numbers is similar in both the RA and non-RA general population. However, higher rates of ASD and revision may still potentially increase surgical complication rates. Strategies, such as those involving better fusion cages and a combination of teriparatide and denosumab therapy after LIFs for the purpose of preventing non-union, have been suggested when discussing the general population [34, 35]. In Taiwan, the control of RA has improved since the increased use of conventional synthetic disease-modifying antirheumatic drugs began in 1990s, as well as the approval for use of both adalimumab and etanercept in 2002 [36]. Therefore, the innovation of anti-inflammatory agents and an interbody fusion technique may result in both better disease control and strength the bone condition for LIFs amongst RA patients.

Limitations surrounding this study included a relatively small sample size and the methodological design of the retrospective study. Moreover, there is no reference to disease activity, length of diagnosis, non-RA control group, or the number and types of anti-inflammatory drug usage, which makes it difficult to draw robust conclusions from the data presented. Subjective measurement bias cannot be easily avoided, even though the operational definition for the parameters was conducted. The FW and FH may be covered by the fused bone and may be disrupted on the plain film. Moreover, since endplate erosion was better visualized through MRI, further studies should be conducted.

## Conclusion

We revealed the radiographic outcomes for symptomatic spinal disease in RA patients with good fusion rates amongst lumbar interbody fusion, including ALIF, OLIF, and TLIF. Despite patients receiving OLIF suffering from a significantly higher rate of cage subsidence, the fusion rate revealed no significant differences when compared to TLIF.

## Data Availability

The original contributions presented in the study are included in the article. Further inquiries can be directed to the corresponding author.

## Abbreviations

ADH: Anterior Disc Height
ALIF: Anterior Lateral Interbody Fusion
APD: Anterior–posterior Diameter
ASD: Adjacent Segment Disease
FH: Foraminal Height
FW: Foraminal Width
LIFs: Lumbar Interbody Fusions
OLIF: Oblique Lateral Interbody Fusion
OP: Operative
PDH: Posterior Disc Height
PLF: Posterolateral Lumbar Fusion
RA: Rheumatoid Arthritis
SL: Segmental Lordosis
TLIF: Transforaminal Lateral Interbody Fusion
WI: Wedge Index
